# A Seroprevalence Study on Residents in a Senior Care Facility with Breakthrough SARS-CoV-2 Omicron Infection

**DOI:** 10.1089/vim.2022.0133

**Published:** 2023-04-14

**Authors:** Heui Man Kim, Eun Ju Lee, Sang Won O, Yong Jun Choi, Hyeokjin Lee, Sae Jin Oh, Jeong-Min Kim, Ae Kyung Park, Jeong-Ah Kim, Chae young Lee, Jong Mu Kim, Hanul Park, Young Joon Park, Jeong-Hee Yu, Eun-Young Kim, Hwa-Pyeong Ko, Eun-Jin Kim

**Affiliations:** ^1^ Division of Emerging Infectious Diseases, Bureau of Infectious Diseases Diagnosis Control, Korea Disease Control and Prevention Agency (KDCA), Cheongju-si, Republic of Korea.; ^2^ Epidemiological Investigation Team, Epidemiological Investigation and Analysis Task Force, Korea Disease Control and Prevention Agency (KDCA), Cheongju-si, Republic of Korea.; ^3^ Honam Regional Center for Disease Control and Prevention, Gwangju-si, Republic of Korea.; ^4^ Gwangju Metropolitan Government, Gwangju-si, Republic of Korea.

**Keywords:** SARS-CoV-2, Omicron variant, breakthrough infection, neutralizing antibody, COVID-19 vaccination, elderly individuals

## Abstract

The Omicron variant of severe acute respiratory syndrome coronavirus 2 (SARS-CoV-2) began spreading rapidly in the community in November 2021, becoming the dominant variant in the Republic of Korea in 2022. Although its pathogenesis in healthy individuals was low, the severity and hospitalization rate was higher in the elderly and immunocompromised patients. We aimed to investigate the immunogenicity in acute and convalescent phases of breakthrough infection by Omicron in elderly individuals. Serological data were assessed by electrochemiluminescence immunoassay, enzyme-linked immunosorbent assay, and plaque-reduction neutralization tests. SARS-CoV-2-specific antibody and immunoglobulin G levels in the acute phase were higher in third dose-vaccinated elderly than in first and second dose-vaccinated patients. The neutralization antibody titer was detected only in third dose-vaccinated patients, and the titer was higher for the Delta than the Omicron variant. In the convalescent phase of Omicron infection, the neutralization antibody titer of vaccinated patients was higher for the Delta than the Omicron variant except in unvaccinated individuals. We demonstrated that the cause of the vulnerability to Omicron variant infection in third dose-vaccinated elderly was due to the low neutralization antibody level against Omicron. A fourth dose of vaccination is required in the elderly to reduce hospitalization and mortality caused by the Omicron variant.

## Introduction

The first case of the Omicron variant infection reported in South Africa in November 2021 was designated as a variant of concern (VOC) by the World Health Organization (WHO) considering multiple mutations in spike regions of the genome leading to fast transmissibility in epidemiological investigations and patients and escape of coronavirus disease 2019 (COVID-19) vaccination (Ao et al., 2022; Araf et al., 2022; Karim and Karim, 2021; Tian et al., 2022). The Omicron variant is currently the dominant severe acute respiratory syndrome coronavirus 2 (SARS-CoV-2) circulating globally, and a variety of sublineages have been classified (Mahase, 2022; Taylor, 2022).

The Omicron epidemic in the Republic of Korea (ROK) was initiated by international travelers in November 2021 and has rapidly spread through the community with a variety of epidemiological linkages, and the detection rate of the Omicron variant has increased since January 2022 (Kim et al., 2022a; Regeneron COVID-19 Dashboard, 2022). The clinical implication of Omicron variant-infected patients is known to be less severe than those of the Delta variant-infected individuals (Kim et al., 2022b; Lauring et al., 2022; Suzuki et al., 2022). However, increased transmissibility of the Omicron variant and evasion of immunity developed by COVID-19 vaccination and previous infection contributed to the worldwide Omicron variant pandemic (Rössler et al., 2022; Torjesen, 2021).

In particular, Omicron variant-infected individuals who were elderly, had underlying diseases, or were immune-deficient showed high disease severity and mortality compared with the healthy young population (Dhawan et al., 2022; Nguyen et al., 2022). Therefore, a fourth booster vaccination was recommended in some countries for the high-risk groups, including elderly and immunocompromised patients, to mitigate their clinical severity and mortality (Bar-On et al., 2022; Burki, 2022). In this study, we investigated the immunogenicity in acute and convalescent phases of breakthrough infection by Omicron through a cohort study on residents in a senior care facility to assess the need for a fourth vaccination in high-risk groups.

## Materials and Methods

### Subjects

The sero-surveillance cohort study was conducted on residents in a senior care facility that reported breakthrough infection in December 2021 in ROK. Participants who received a third (*n* = 66), second (*n* = 3), and first dose (*n* = 4) of the vaccine as well as unvaccinated (*n* = 4) individuals were recruited. The median age of the participants was 80.1 (49–100) years, and the age of 80 was the most distributed (44.3%) in the cohort.

### Clinical specimens

Blood samples were obtained from participants infected with SARS-CoV-2 Omicron variant (confirmed by whole genome sequencing) in acute phase (within 3 days of post-symptom onset) and convalescent phase (after 14 from 21 days of post-symptom onset) (Park et al., 2021). Institutional Review Board approval was obtained from the Korea Disease Control and Prevention Agency (2021-12-02-PE-A).

### Electrochemiluminescence immunoassay

Quantitative Elecsys Anti-SARS-CoV-2 S assay (Elecsys Anti-S; Roche Diagnostics, Mannheim, Germany) and qualitative Elecsys Anti-SARS-CoV-2 assay (Elecsys Anti-N; Roche Diagnostics) were used for immunoassay. The results were measured on a cobas e 602 module (Roche). All samples were processed according to the manufacturer's instructions.

### SARS-CoV-2 immunoglobulin G enzyme-linked immunosorbent assay

SARS-CoV-2 antibody assay (AdvanSure SARS-CoV-2 immunoglobulin G [IgG]; LG Chem, Seoul, Korea) was performed using the Epoch Microplate Spectrophotometer and ELx50 Filter Microplate Washer (BioTek Instruments, Winooski, VT). The assay was carried out according to the manufacturer's instructions.

### Plaque-reduction neutralization assay

The heat-inactivated plasma samples were fivefold serially diluted in culture medium with a starting dilution of 1:10. The diluted plasma was mixed with 1,000 PFU/mL of SARS-CoV-2 Omicron (BA.1.1) and Delta (AY.69) variants. After 1 h of incubation at 37°C, the plasma and virus mixtures were inoculated onto 12-well plates with a monolayer of Vero E6 cells preseeded the previous day and then cultured for 2–3 days. Plaques and the dilution of sera that showed 50% inhibition were counted. The neutralization titer (PRNT_50_) of the test plasma sample is defined as the reciprocal of the highest dilution of plasma required for a 50% reduction in infection plaque compared with the plaque counts in the virus-only control.

### Statistical analysis

Unpaired Mann–Whitney *U* test was applied to compare immunogenicity of the participants according to the number of vaccinations or SARS-CoV-2 infections. *p*-Values ≤0.05 indicated significance.

## Results

### Study cohort

This study was conducted on 96 patients of a senior care facility in Korea. One NP antibody-positive patient in the acute phase and 18 patients from whom paired serum samples were not collected in the acute and convalescent phases were excluded. The remaining 77 patients confirmed positive for SARS-CoV-2 infection were classified as unvaccinated (*n* = 4) and vaccinated with the first (*n* = 4), second (*n* = 3), and third dose (*n* = 66). The patients were vaccinated with AZD1222 or BNT162b2 or boosted with BNT162b2 after AZD1222 vaccination. The average elapsed days after the last vaccination was 257.50 days (interquartile range [IQR]: 179.0–303.0) for those vaccinated with the first dose, 151.67 days (IQR: 78.0–258.0) with the second dose, and 62.18 days (IQR: 57.80–73.0) with the third dose (Table 1).

**Table 1.** Participants Included in this Study

Groups	COVID-19 confirmed cases	Median age in years	Gender (female/male)	Vaccine information	Days post-last vaccination
Unvaccinated	4	84.5	4/0	NA	NA
Vaccinated with first dose	4	76	2/2	AZD1222 (*n* = 2), BNT162b2 (*n* = 2)	257.5
Vaccinated with second dose	3	74.7	1/2	AZD1222+AZD1222 (*n* = 2), BNT162b2+BNT162b2 (*n* = 1)	151.7
Vaccinated with third dose	66	80.4	44/22	AZD1222+AZD1222+BNT162b2 (*n* = 62), BNT162b2+BNT162b2+BNT162b2 (*n* = 4)	62.2
Total	77	80.1	51/26	NA	NA

AZD1222 and BNT162b2 represent COVID-19 vaccines manufactured by AstraZeneca and Pfizer BioNTech, respectively.

COVID-19, coronavirus disease 2019; NS, not applicable.

### Quantitative SARS-CoV-2 Spike-specific antibodies

**FIG. 1.**
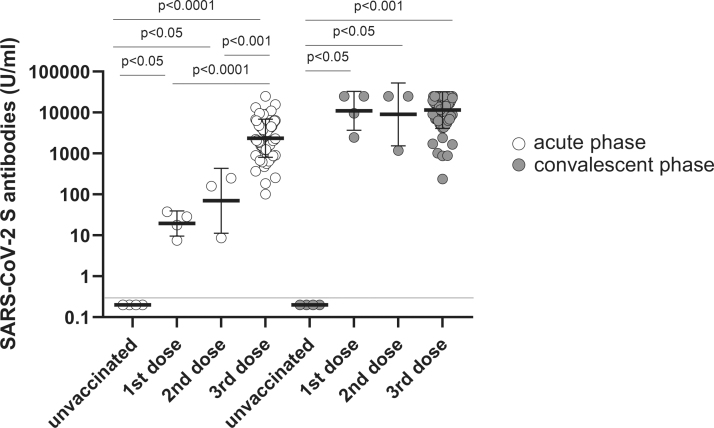
SARS-CoV-2 S-specific antibody was elicited by COVID-19 vaccination and breakthrough infection (Omicron variant) in elderly individuals. The quantitative SARS-CoV-2 Spike-specific antibody was measured in elderly unvaccinated (*n* = 4) and vaccinated individuals with first (*n* = 4), second (*n* = 3), or third dose (*n* = 66) in acute and convalescent phases. The individual shapes represent geometric mean titers from two independent experiments consisting of two replicates each. Lines represent geometric means and error bars represent geometric standard deviations for each group. The corresponding cutoff was >0.4 U/mL (red line) for the quantitative Elecsys Anti-SARS-CoV-2 S assay. The empty and solid circles represent acute and convalescent phases, respectively. SARS-CoV-2, severe acute respiratory syndrome coronavirus 2.

All the vaccinated elderly individuals produced the antibody in the acute phase, whereas the unvaccinated elderly individuals were nonreactive (<0.4 U/mL). The antibody level increased with increase in the number of vaccinations. The average level of antibody was 19.53 U/mL (IQR: 10.11–35.45) in those vaccinated with the first dose, 70.0 U/mL (IQR: 8.63–250.0) in those vaccinated with the second dose, and >2,360.0 U/mL (IQR: 1,292.0–4,703.0) in those vaccinated with the third dose during the acute phase. In the convalescent phase, although the antibody was not detected in unvaccinated COVID-19 cases, the antibody level in the vaccinated groups significantly increased to 11,026.0 U/mL (IQR: 4,248.0–25,000.0) in those vaccinated with the first dose, 9,030.0 (IQR: 1,178.0–25,000.0) in those vaccinated with the second dose, and 11,528.0 (IQR: 6,862.0–25,000.0) in those vaccinated with the third dose (Fig. 1).

### Quantitative SARS-CoV-2-specific IgG

**FIG. 2.**
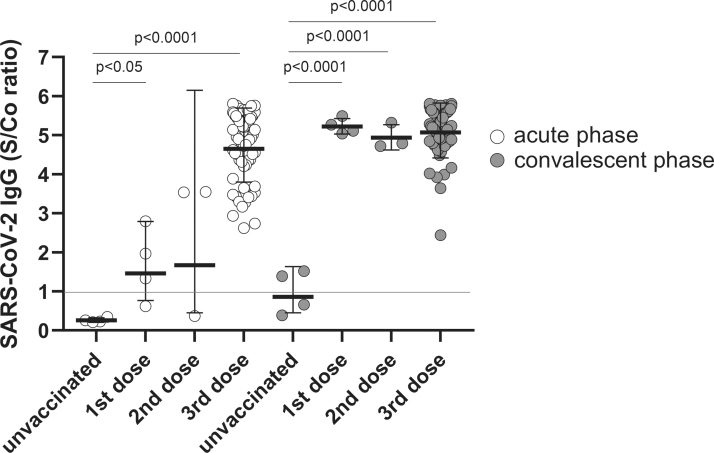
SARS-CoV-2 (S1)-specific IgG was elicited by COVID-19 vaccination and breakthrough infection (Omicron variant) in elderly individuals SARS-CoV-2 (S1)-specific IgG was measured in elderly unvaccinated (*n* = 4) and vaccinated individuals with first (*n* = 4), second (*n* = 3), or third dose (*n* = 66) in acute and convalescent phases. Individual shapes represent geometric mean titers from two independent experiments consisting of two replicates each. Lines represent geometric means and error bars represent geometric standard deviations for each group. The corresponding cutoff was >1.0 S/Co ratio (red line) for SARS-CoV-2 (S1) IgG enzyme-linked immunosorbent assay. The empty and solid circles represent acute and convalescent phases, respectively. COVID-19, coronavirus disease 2019; IgG, immunoglobulin G; S/Co, signal/cutoff.

IgG levels were significantly increased only in those vaccinated with the first dose and booster in the acute phase. The geometric mean titer in those vaccinated with third dose (GMT 4.65 signal/cutoff [S/Co] ratio, IQR: 4.30–5.43) was 3.18-fold higher than in those vaccinated with the first dose (GMT 1.46 S/Co ratio, IQR: 0.80–2.59). The IgG titer was positive even in the convalescent phase and was significantly elevated in the vaccinated groups (first, second, and third doses), unlike in the unvaccinated groups. The IgG titer was GMT 5.23 S/Co ratio (IQR: 5.09–5.44) in first dose-vaccinated patients, GMT 4.94 (IQR: 4.72–5.32) in third dose-vaccinated patients, and GMT 5.07 (IQR: 4.84–5.58) in the convalescent phase (Fig. 2).

### Neutralization antibody against Omicron or Delta variants

**FIG. 3.**
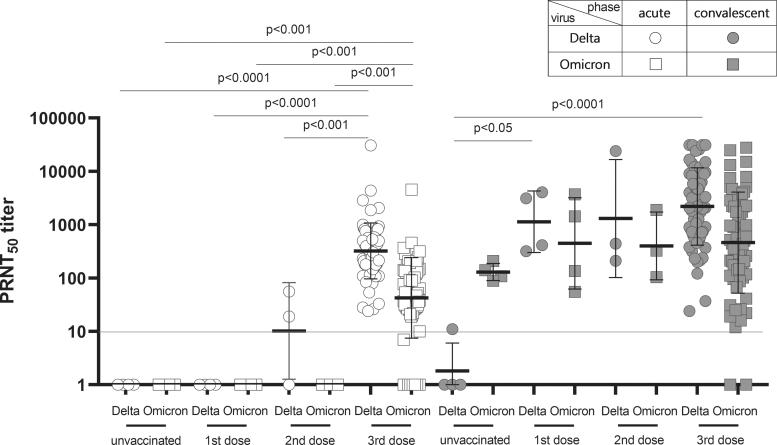
Neutralization antibody titer against Omicron was insufficient in elderly vaccinated individuals with a third dose, and the titer was boosted in the convalescent phase in all the vaccinated elderly individuals, whereas the neutralization antibody titer was higher against the Delta variant than the Omicron variant in vaccinated elderly individuals and vice versa in unvaccinated elderly individuals. The neutralization antibody titer against Delta and Omicron variants was measured in elderly unvaccinated (*n* = 4) and vaccinated individuals with first (*n* = 4), second (*n* = 3), or third dose (*n* = 66) in acute and convalescent phases. Individual shapes represent geometric mean titers from two independent experiments consisting of two replicates each. Lines represent geometric means and error bars represent geometric standard deviations for each group. The corresponding cutoff was >10 PRNT_50_ (red line) for the plaque reduction neutralization test. Circle and square represent Delta and Omicron variants used in the plaque reduction neutralization test, respectively. The empty and solid shapes represent acute and convalescent phases, respectively.

The patients vaccinated with the third dose possessed the calculated neutralization antibody titer against Delta and Omicron variants in the acute phase. The titer for the Delta variant (GMT 322.20 PRNT_50_, IQR: 157.30–583.80) was 7.59-fold higher than that for the Omicron variant (GMT 42.47 PRNT_50_, IQR: 26.75–136.80). Although the titer was detected in second dose-vaccinated patients, the titer was on the border line. In the convalescent phase, the titer was increased in all the vaccinated elderly individuals unlike in the acute phase. However, the highest titer was detected in third dose-vaccinated patients, which was measured as GMT 2197.0 PRNT_50_ (IQR: 637.30–8,959.0) for the Delta variant and GMT 461.0 PRNT_50_ (IQR: 134.30–2,421.0) for the Omicron variant (Fig. 3).

However, the titer was not elicited for the Delta variant in unvaccinated cases, even in the convalescent phase. As the number of vaccination applications increased, the neutralization antibody titer was increased in the convalescent phase. The level of titer was as follows: GMT 1,136.0 PRNT_50_ (IQR: 341.50–3,832.0) for first dose-vaccinated elderly individuals, GMT 1,306.0 PRNT_50_ (IQR: 210.0–24,037.0) for second dose-vaccinated elderly patients, and GMT 2,197.0 PRNT_50_ (IQR: 637.30–8,959.0) for third dose-vaccinated elderly individuals against the Delta variant. However, no significant difference was observed in the titer against the Omicron variant among the first (GMT 446.70 PRNT_50_, IQR: 75.25–3,142.0), second (GMT 400.60 PRNT_50_, IQR: 104.0–1,890.0), and third dose-vaccinated (GMT 461.60 PRNT_50_, IQR: 134.30–2,421.0) patients (Fig. 3).

## Discussion

Serological studies are critical for understanding the characterization of the newly emerged VOCs of SARS-CoV-2 (Chmielewska et al., 2021). In this study, we investigated the humoral immune response in patients in a senior care facility who were infected with the Omicron variant. Despite the high COVID-19 vaccination rate (94.8% for over first dose vaccinated and 85.7% for those who received a third dose), the patients were infected with the Omicron variant. It is known that the elderly are more vulnerable to VOCs of SARS-CoV-2 than healthy adults due to reduced magnitude and durability of humoral immune responses related to underlying health conditions, even if they were vaccinated with a third dose (Brockman et al., 2021; Romero-Olmedo et al., 2022).

This seroprevalence study was conducted only on the elderly with confirmed COVID-19 infection, and there is a limitation in that there was a lack of serological data for healthy adults. However, the neutralization antibody titers between serum only for acute phase elderly vaccinated cases were compared with a third dose to exclude immunity due to the natural infection and serum for healthy adults vaccinated with a third dose unrelated to this case. The neutralization antibody titer of health care workers was 4.0-fold and 10.10-fold significantly higher than elderly individuals against the Delta and Omicron variants, respectively (Supplementary Fig. S1). Through the measurement of anti-Spike antibody or specific IgG for SARS-CoV-2, it was possible to confirm COVID-19 vaccination and increased humoral immunity according to number of the vaccinations in acute phase.

However, interestingly, it was impossible to detect the humoral immunity in convalescent phase only for unvaccinated elderly individuals. It is presumed that anti-Spike antibody and S1-specific IgG manufactured based on a prototype of SARS-CoV-2 were not detected in unvaccinated elderly individuals infected with the Omicron variant due to many mutation sites of the spike gene in the Omicron variant. However, all unvaccinated (*n* = 4) elderly individuals showed seropositivity for anti-N (nucleocapsid) in convalescent phase.

Therefore, to evaluate the immunogenicity of an updated vaccine, it is necessary to consider applying an antibody assay designed for the recent variants of SARS-CoV-2. In this study, we demonstrated that the cause of the vulnerability to the Omicron variant infection in third dose-vaccinated elderly individuals was due to the low neutralization antibody levels against the Omicron variant through the plaque reduction neutralization test. These results support the consideration of a fourth dose vaccination in the elderly to reduce hospitalization and mortality caused by the Omicron variant.

## Supplementary Material

Supplemental data

## Data Availability

The data generated in this study are present in the main text or in the Supplementary Materials.
