# Supplementary material for: A Seroprevalence Study on Residents in a Senior Care Facility with Breakthrough SARS-CoV-2 Omicron Infection
Source: Viral Immunol. 2023 Apr 14;36(3):203–8. doi: 10.1089/vim.2022.0133 (PMC10621659; doi:10.1089/vim.2022.0133)

**Supplemental Figure 1. Neutralization antibody titer of healthy adults was higher than that of elderly individuals against both Omicron and Delta variants.**

The neutralization antibody titer against the Delta and Omicron variants was measured in elderly vaccinated individuals with a 3^rd^ dose (n = 66) and healthcare workers vaccinated with a 3^rd^ dose (n = 10). For the elderly individuals infected with the Omicron variant, we used serum collected in the acute phase (62.3 mean days of post last vaccination). The immunized serum (41.0 mean days post-last vaccination) was used from healthcare workers vaccinated with a 3^rd^ dose without COVID-19 infection.

Individual shapes represent geometric mean titers form two independent experiments consisting of two replicates each. The lines represent geometric means, and the error bars represent geometric standard deviations for each group. The corresponding cut-off was > 10 PRNT_50_ (red line) for the plaque reduction neutralization test. Circle and square represent elderly individuals and healthcare workers, respectively. The empty and solid shapes represent the Delta and Omicron variants used in the plaque reduction neutralization test, respectively.


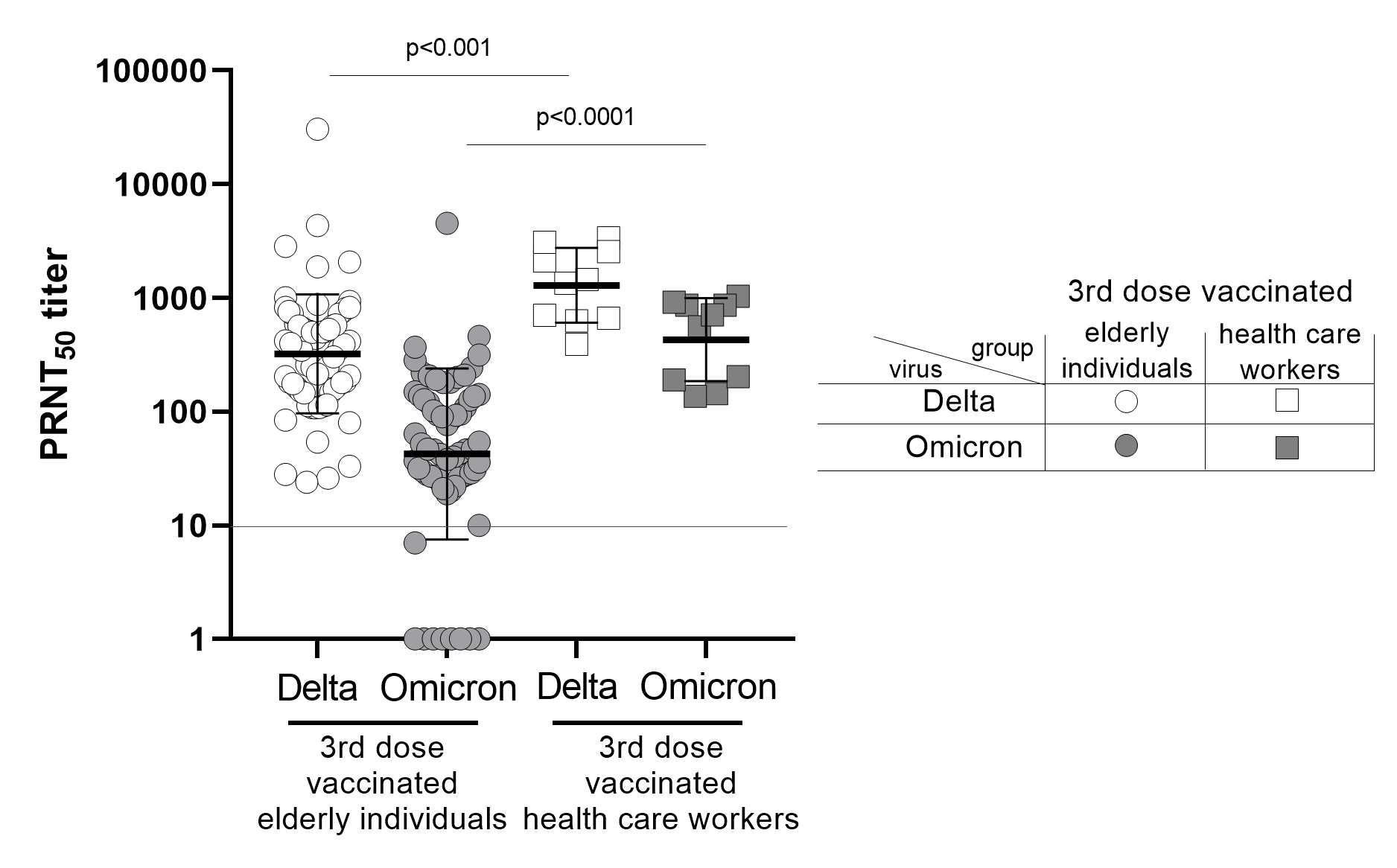

Supplement: Supplemental data [file Suppl_FigureS1.docx]
